# Preferential expression of hPGFS in primary SCCHN and tumour cell lines derived from respiratory and digestive organs

**DOI:** 10.1038/sj.bjc.6601636

**Published:** 2004-03-02

**Authors:** S Li, E Hanna, R Breau, V Ratanatharathorn, X Xia, J Suen

**Affiliations:** 1 Department of Comparative Biomedical Sciences, SVM, Louisiana State University, Skip Bertman Drive, LA 70803, USA; 2 Department of Otolaryngology/Head and Neck Surgery, University of Arkansas for Medical Sciences, 4301 West Markham, Slot 543, Little Rock, AR 72205, USA; 3 Department of Radiation Oncology, University of Arkansas for Medical Sciences, 4301 West Markham, Slot 771, Little Rock, AR 72205, USA

**Keywords:** differential display, head and neck cancer, colon cancer, lung cancer, gene expression, hPGFS

## Abstract

Identifying overexpressed genes in tumours is a critical step for tumour diagnosis, prognosis, and treatment. Using differential display polymerase chain reaction, sequence analysis, and gene Blast searches, we discovered that human prostaglandin F synthase (*hPGFS*) was upregulated in squamous cell carcinoma of the head and neck (SCCHN). Northern blot analysis indicated that up to a 16-fold increase in the level of hPGFS expression was detected in 40.5% (15 out of 37) of SCCHN primary tumours. The increased expression of hPGFS in SCCHN was primarily detected in SCC of larynx and hypopharynx (59%, *P*<0.05). Using the same primary tissue samples, increased levels of epidermal growth factor receptor (EGFR) expression were detected in only 32% of tumour tissues, suggesting hPGFS may have the potential to become a drug target or molecular marker for SCCHN. To determine if the increased level of hPGFS expression came from tumour cells, we determined the level of hPGFS expression in SCCHN tumour cell lines. A high level of hPGFS expression was detected in four out of five tumour SCCHN cell lines. To determine if upregulation of hPGFS is SCCHN-specific, hPGFS expression was analysed in 59 tumour cell lines derived from different types of tumours. The expression of hPGFS was increased from two- to 500-fold in a large portion of cell lines derived from lung (five out of nine), colon (five out of seven) as well as head and neck cancer (four out of five). These data link hPGFS expression to tumours located in the respiratory and digestive organs.

Squamous cell carcinoma of the head and neck (SCCHN) is the fourth most common malignancy among males in the US, with more than 40 000 cases diagnosed each year. In Europe, 60 000 cases are diagnosed yearly, compared to 500 000 cases worldwide (Chuaqui *et al*, 1997; Chan *et al*, 1998). Only a few biomarkers and therapeutic gene targets are known to display alterations in SCCHN. These include the commonly known markers for other tumours, such as *p53*, *p53Ab*, *p16*, *Rb*, *PTEN*, *EGFR*, *bcl2*, *p21*, and *VEGF* (Koch, 1999). Use of proliferation markers, such as Ki-67, and proliferating cell nuclear antigen for diagnosis of SCCHN has also been attempted, but they are not tumour-specific. To design better strategies for targeting gene therapy, as well as for diagnosis and prognosis, it is important not only to validate the existing genes but also to discover novel genes for SCC (Sehgal *et al*, 1998). This is also true for other types of tumours.

Since its invention in 1992, the DD technique has evolved into a powerful method for elucidating changes in gene expression in response to disease development and treatment, as well as studying gene functions and cloning novel genes (Liang and Pardee, 1992; Liang *et al*, 1992; Sun *et al* 1994; Lehar *et al*, 1996). Differential display allows the mRNA profile of all genes expressed from two different samples to be compared side by side, on the same gel, making the simultaneous detection of both up- and downregulated gene expression possible (Liang *et al*, 1992). This technique has been widely used in studying up- or downregulated genes in many tumour tissues, including prostate, thyroid (Musholt *et al*, 1997), pancreatic (Ozaki *et al*, 1998), and breast cancers (Watson and Fleming, 1994), colorectal and oesophageal carcinomas (Graber *et al*, 1996; Chan *et al*, 1998), melanoma (Duncan *et al*, 1998), and glioblastoma (Chuaqui *et al*, 1997; Sehgal *et al*, 1997). However, to our knowledge, no study using this technique in primary SCCHN tissues has been performed, although a few studies have reported using mouse or tumour cell lines of SCCHN (Gorogh *et al*, 1997; Patel *et al*, 1997; Gottschlich *et al*, 1999; Dong *et al*, 2001).

Here, we report the discovery of three genes differentially expressed in SCCHN tissues. The most interesting gene is human lung prostaglandin F synthase (*hPGFS*) that was increased in more than 59% of larynx or hypopharynx SCC tumour tissues, compared to matched surgical margins. The increased expression of hPGFS was also detected in most of the tumour cell lines (55–80%) derived from lung, colon, and head and neck cancer, but in a small portion of tumour cell lines of leukaemia, breast, central nervous system, renal, and ovary cancer. prostaglandin F synthase was not detected in normal mucosal cell lines and melanoma cell lines. Thus, the magnitude of increase in hPGFS expression seems dependent on tumour types.

## MATERIALS AND METHODS

### Cell culture and tissue specimen

SCCHN cell lines were obtained from the laboratories of Teresa Whiteside, PhD (University of Pittsburgh, PA, USA) and Tom Carey, PhD (University of Michigan, Albany, USA), and Normal mucosal cell lines (NCM356, NCM425, and NCM 469) were obtained from Incell Co, LLC (San Antonio, TX, USA). All cells were grown in AIM-V medium supplemented with 10% foetal calf serum, except for normal mucosal cell lines, which were grown in a M13 medium (Incell Co., LLC). Normal mucosa, surgical margins, and primary SCC tissues were obtained from 37 patients undergoing surgical resection of previously untreated SCCHN at the University of Arkansas for Medical Sciences (UAMS). After surgical removal, samples were immediately snap-frozen in a liquid nitrogen container. All surgical margins used in this study were determined to be pathologically negative. This protocol was approved by the UAMS Institutional Review Board.

### Differential display

**Figure 1:**
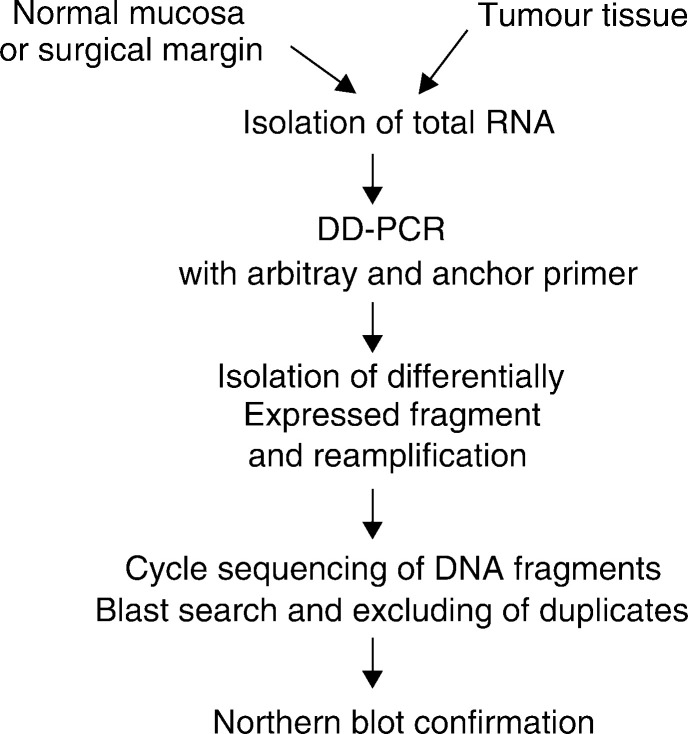
Outline of a modified differential display process for isolating differentially expressed genes from SCCHN tissue.

For DD polymerase chain reaction (DD-PCR), normal mucosa, negative surgical margins, and the matching tumour tissues were homogenised with a bead-beater in TRIzol reagent (Life Technologies, Inc., Rockville, MD, USA), as described previously (Li *et al*, 1999), to extract total RNA. Total RNA (30 *μ*g) was treated with 10 U of DNase I (MessageClean Kit, GenHunter Corp., Nashville, TN, USA) for 30 min at 37°C to digest any DNA contaminants. The flow chart for the procedure is outlined in Figure 1

. Reverse transcriptase (RT)–PCR was performed using the following oligonucleotide primers: RT primer 5′TTTTTTTTTTTN 3′ (T_11_N; N=G,C,A) and arbitrary 10-mer primer (AP1-8) (GenHunter Corp., Nashville, TN, USA). Total RNA (200 ng) was reverse-transcribed using 50 U of Moloney murine leukaemia virus RT in the presence of one RT primer (T_11_N) and 20 *μ*mol of deoxyribonucleoside triphosphate (dNTP) for 5 min at 65°C, followed by 60 min at 37°C. After heat inactivation of the RT at 95°C for 5 min and subsequent cooling to 4°C, 2 *μ*l of the cDNA samples were added to 18 *μ*l of the PCR reaction mixture, containing 200 nM of one of the eight arbitrary 10-mer primers AP1-8 and T_11_N, 0.2 *μ*l of *α*^33^P-dATP (2000 Ci mM^−1^; NEN Life Science Products, Inc., Boston, MA, USA), and 0.2 *μ*l of Taq DNA polymerase. The PCR parameters during 40 cycles were 94°C for 30 s, 40°C for 2 min, and 72°C for 30 s. Finally, the samples were heated to 72°C for 5 min and then cooled to 4°C. The PCR products were analysed on 6% DNA sequencing gels and exposed on an X-ray film. Bands of interest were cut from the dried gels and boiled in a TE (tris-EDTA) buffer to extract the DNA. The extracted DNA was further amplified by PCR using the same set of primers. The amplified DNA was subjected to agarose-gel electrophoresis, and the DNA was purified from the gel with the use of a gel extraction kit (Qiagen, Inc., Valencia, CA, USA). The recovered DNA samples were subjected to sequencing with the matching T_11_N anchoring primer, using a cycle sequence technique in the presence of a radiolabeled terminator (USB Corp., Cleveland, OH, USA). The recovered DNA was also used as template to make probes for Northern blot analysis as described below.

### Northern blot analysis

The technique used for Northern blot analysis was the same as that described previously (Li *et al*, 1997). The extracted fragments that were amplified by the PCR technique were used as probes for Northern blot analysis. The probes were labelled with *α*^32^P-dCTP (3000 Ci mM^−1^, Amersham Pharmacia Biotech, Piscataway, NJ, USA) using Strip-EZ™ PCR kit (Ambion, Inc., Austin, TX, USA, USA). The RNA of SCCHN and normal mucosal cells were isolated from our own cell culture by the methods described above. Approximately 25 *μ*g of total RNA from tissues and 10 *μ*g from cell lines were subjected to 1% agarose-formaldehyde gel electrophoresis at 60 V for 3 h. The RNA was transferred to a positively charged nylon membrane (Boehringer Mannheim, Indianapolis, IN, USA) and prehybridised and hybridised at 42°C in a buffer purchased from Ambion. Hybridisation of the same filters with a probe for *β*-actin was used as an internal control for RNA loading, and methylene blue staining was used to determine the relative sizes of hybridised RNA (Li *et al*, 1997). The Northern blot results were quantified by scanning the expression signal intensity with a PhosphorImager analyser (Model 445 SI, Molecular Dynamics, Sunnyvale, CA, USA).

### Statistical analysis of hPGFS expression

The level of hPGFS expression between normal and tumour tissues of larynx, hypopharynx, or overall sites was determined with paired *t*-test. Statistical significance was defined as a *P*<0.05.

## RESULTS

### Determination of the appropriate controls for discovery of novel tumour-related genes using DD-PCR and isolation of SCCHN-related gene fragments

**Figure 2:**
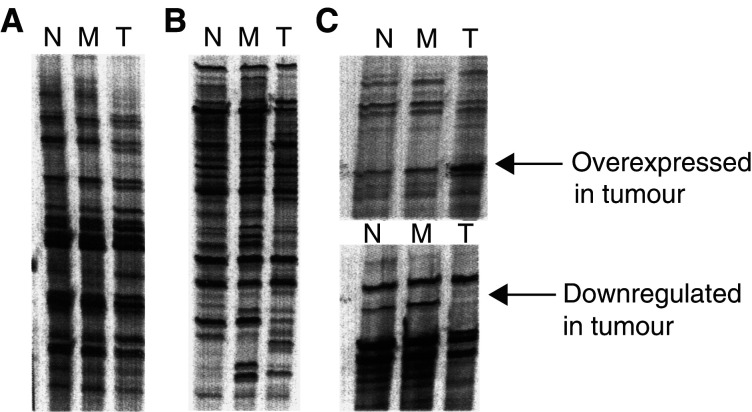
Identification of differentially expressed genes from SCCHN tissue, normal mucosa (M), and negative surgical margin (N) with the use of DD-PCR as described in the Materials and Methods section. (**A**, **B**) Autoradiogram of DD-PCR using different 5′ arbitrary 10-mer primer and one 3′ primer (T_11_N) (N=C,G, A). (**C**) Autoradiogram to highlight the gene fragments, which are either overexpressed or downregulated in tumour tissue, cut out of the gel.

One of the most critical steps in the DD-PCR analysis was the determination of a reliable control. Although this was a difficult task with cultured cell lines, it was even more challenging when primary tumour tissues were used. To determine the appropriate control tissue to perform DD-PCR for isolation of the tumour-specific gene from SCCHN, we tested normal mucosa, skin, and negative surgical margins. Our primary analysis, using T_11_N (N=C,G,A) and an arbitrary 10-nucleotide primer, indicated that the expression profile in skin tissue was totally different from the profiles of normal mucosa, the tumour surgical margin, and the tumour. On the other hand, both normal mucosa and negative surgical margins demonstrated a similar gene expression profile (Figure 2A and B

), indicating either surgical margin or normal mucosa can effectively serve as negative controls for DD-PCR analysis. Thus, both of them were used as controls. Upon comparison of the gene expression profile of tumour tissue with those of surgical margins and normal mucosa, approximately 24 differentially expressed DNA fragments were identified by DD-PCR (Figure 2C, Table 1

**Table 1:** Isolation of tumour-associated genes by DD-PCR and confirmation by Northern blot analysis

**Name**	**Primers**	**Expression in tumour by DD-PCR**	**Genes**	**Accession number**	**Size of mRNA**	**Ratio of T/N by NB**
A1-1	AP1,T11A	Down	sFRP	NM003012	4.5 kb	0.2
A4-1	AP4,T11A	Down	BENE	NM005434	2.8 kb	NC
A4-5	AP5,T11A	Down	Unknown	AC008149	1 kb	ND
A4-6	AP6,T11A	Down	DSG3	NM001944	7 kb	0.3
A6-3	AP3,T11A	Down	Unknown	AC161645	6 kb	NC
A6-2	AP2,T11A	Down	Unknown	AC008513	ND	NA
G4-1	AP4,T11G	Down	Unknown	AC013391	ND	NA
G4-2	AP2,T11G	Down	Unknown	AC016738	ND	NA
G4-5	AP5,T11G	Down	TGM3	AC031678	1 kb	NC
C2-1	AP1,T11C	Down	CILP	AF035455	1 kb	NC
C3-1	AP1,T11C	Down	RTP	D87953	4 kb	NC
C5-1	AP1,T11C	Down	EHHADH	L07077	2.5 kb	IC
C6-1	AP1,T11C	Down	E2	X56832	7.6 kb	NA
A3-3	AP3,T11A	Up	P54NRB	Y11298	2.6 kb	NA
A4-4	AP4,T11A	Up	hPGFS	AB018580	1.2 kb	16.1
G4-3	AP3,T11G	Up	Unknown	AC087882	ND	NA
G4-7	AP7,T11G	Up	PIGR	P01833	4.3 kb	IC
G6-1	AP1,T11G	Up	Unknown	AC357972	ND	NA
C4-1	AP1,T11C	Up	Unknown	AC139004	ND	NA

NB=Northern blot analysis; T/N=average expression ratio in two tumours *vs* in two surgical margins; ND=not determined; NC=no change in expression; IC=inconsistent in expression; NA=not available; DD-PCR=differential display-polymerase chain reaction.

). These DNA fragments were isolated directly from the dried gel as summarised in Table 1.

### Identification of the isolated DNA fragments by sequencing and Northern blot analysis

**Figure 3:**
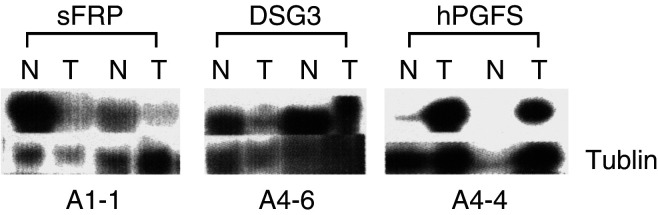
Northern blot analysis of three differentially expressed genes in SCCHN tissues and the matching negative surgical margins. The purpose of this study is to determine the expression of sFRP, DSG3, and hPGFS in two pairs of tumours (T) and negative surgical margins (N). The gene probes were isolated from differential display dried gels, and subjected to sequence analysis. The names of gene fragments were defined by BLAST search from the gene bank at the National Cancer Institute (NCI). Actin was used as an internal control.

We had the option to exclude the false-positive fragments by performing Northern blot analysis, first. Instead, we performed sequence analysis of these fragments because the RNA samples from primary tumour tissues were extremely hard to obtain, and Northern blot analysis needed a relatively large amount of RNA for each gene screening. We found duplicates for five out of 24 fragments, using BLAST search from the gene bank. Another time-saving modification of this procedure was using the isolated DNA fragments for direct sequence analysis instead of cloning prior to sequencing. According to the sequence analysis, the majority of the genes were unknown. The sizes of their cDNA were identified by Northern blot analysis (Table 1), using the isolated DNA fragments as probes. Among the 19 fragments, three were differentially expressed in two separate pairs of tissues used for DD (Figure 3

). The rest either showed no change in gene expression or showed change in one pair but not in the other pair of tissues (data not shown). Among the three genes that were differentially expressed on a uniform basis, two were downregulated and one upregulated. The two downregulated genes in tumours were *sFRP* and desmogelein 3 (*DSG3)*; the upregulated gene was identical to the sequence of *hPGFS* (Suzuki-Yamamoto *et al*, 1999) and *KIAA0119* (Nagase *et al*, 1995), according to the BLAST search from gene bank. As downregulation of secreted Frizzled-related protein (sFRP) has been reported to be tumour-related (Finch *et al*, 1997), we did not further characterise this gene. Since the *DSG3* cDNA is 7 kb and it was difficult to obtain a high-quality Northern blot result from the total RNA isolated from tumour tissues, we plan to characterise it in the future using a different method. A significant difference existed in the level of hPGFS expression between tumour tissues from surgical margins, as determined by Northern blot analysis (Figure 3).

### Expression of hPGFS in the SCC primary tissues

To further demonstrate that hPGFS expression is elevated in SCCHN tumours, we determined the level of hPGFS expression using Northern blot analysis in 37 pairs of SCCHN and surgical margin samples (Table 2

**Table 2:** hPGFS and EGFR expression in tumours and surgical margins

**Samples**	**T**	**N**	**M**	**Tissue origion**	**hPGFS (T/N)**	**EGFR (T/N)**
X19-2	1	n/a	0	Larynx/glottis	16	2.9
X22-9	4	2b	0	Oral cavity/FOM-ant	15	5.4
X19-11	2	n/a	0	Nasal SCC	10	No diff
X23-1	1	n/a	0	Oral cavity/lateral tongue	10	No diff
X13-14	3	0	0	Larynx/glottis	9.6	1.5
X26-3					8.4	3.3
X16-3	4	2c	0	Larynx/glottis	7.3	1.6
X14-4	4	0	0	Hypo/piri	7	No diff
X24-9	2	0	0	Larynx/supraglottis	6	LE
X22-8	2	1	0	Hypopharynx/pyriform	4	2.7
X19-14	2	n/a	0	Hypopharynx/pyriform	3.5	No diff
X14-2	4b	0	0	Maxillary sinus	2.7	No diff
X19-1	2	n/a	0	Larynx/supraglottis	2.6	1.9
X19-7	4	2c	0	Larynx/supraglottis	2.2	No diff
X23-3	3	3	0	Larynx/glottis	2	No diff

X14-5	4	2b	0	Oral cavity/palette	No diff	No diff
X16-1	3	n/a	0	Oral pharynx/bot	No diff	No diff
X19-10	4	2b	0	Oral cavity/FOM	No diff	0.4
X19-12	n/a	1	0	Larynx/glottis	No diff	1.7
X19-13	1	n/a	0	Larynx/glottis	No diff	No diff
X19-15	1	0	0	Oral cavity/tongue	No diff	No diff
X19-3	2	0	0	Oral cavity/FOM	No diff	1.7
X19-4	3	0	0	Larynx/glottis	No diff	2.3
X19-9	4	n/a	0	Oral cavity/palette	No diff	No diff
x22-4	4	3		n/a	No diff	No diff
X22-6	3	2b	0	Oral cavity/FOM	No diff	No diff
X23-10	2	0	0	Oral cavity/lateral tongue	No diff	No diff
X23-2	3	1	0	Oral pharynx/tonsil	No diff	No diff
X23-4	2	1	0	Oral cavity	No diff	2.5
X23-5	2	3	0	Oral cavity/tongue	No diff	No diff
X24-3	n/a	n/a	n/a	Oral cavity	No diff	No diff
X24-5	2	n/a	n/a	Oral cavity	No diff	No diff
X24-6	2	n/a	0	Paranasalsinus/max	No diff	align="center"No diff
X24-7	3	0	0	Hypopharynx/pyriform	No diff	No diff
X26-5					No diff	No diff

X24-2	n/a	2b	0	Larynx/glottis	0.5	No diff
X26-4	n/a	n/a	n/a	n/a	0.1	4.4

T/N, the ratio of gene expression in the tumour *vs* in the matched surgical margin.

). As demonstrated, the level of hPGFS expression was increased in 15 out of 37 SCCHN tumour tissues. Interestingly, the upregulation of hPGFS was detected in 10 out of 17 primary SCC tumours from larynx and hypopharynx (59%, *P*<0.05), but in only a couple of oral SCC (Table 2). Increased expression of hPGFS was independent of tumour stage.

### Expression of epidermal growth factor receptor (EGFR) in the primary tissues derived from surgical margins and tumours

To determine whether the increased level of hPGFS expression correlates to the expression of a well-known tumour marker and drug target, EGFR. The same Northern blot membranes used for analysing hPGFS expression were hybridised with the EGFR probe. As shown in Table 2, 12 out of 37 tumour samples demonstrated more than a 1.5-fold increase in the level of EGFR expression. There is no direct correlation between EGFR and hPGFS expression. However, a high frequency (46.7%) of elevated expression of EGFR was found in the tumours in which increased expression of hPGFS was also found; a low frequency (22.7%) of elevated EGFR expression was found in the tumours in which hPGFS expression was not increased or decreased (Table 3

**Table 3:** hPGFS expression in tumour cell lines

**Tissue origion**	**Cell lines**	**Level of increase**	**Cell lines**	**Tumours**	**Level of increase**
Normal mucosa	NCM356	0.8	Melanoma	RPMI7951	1.4
	NCM425	1.0		Malme3M	1
	NCM460	1.5		LoxIMVI	0.6
				UACC62	0.4
SCCHN	UMSCC38	74.5		UACC257	0.2
	PCI52	67.2		SK-MEL5	0.2
	PCI1	8.6		SK-MEL28	0.2
	UMSCC2	9.5		SK-MEL-2	0.6
				M14	0.2

Colon cancer	COLO205	105	Leukemia	K562	6.6
	HCC2998	32.8		CCrf-Cem	1.4
	HT29	5.9		MOLT4	0.8
	KM126	2.1		SR	0.4
	SW620	6.2		HL60	0.4
	HCT15	0.8			
	HCT116	1.7			

Non-small-cell lung cancer	A549	500.4	Breast cancer	MCF7	4.6
	NCI-H226	123.4		MDA-MB231	0.4
	NCI-H460	66.7		MDA-MB469	0.4
	NCI-H23	9.8		NCI/ADR-RES	0.4
	EKVX	8.4		HS578T	0.4
	HOP-92	1.0		BT549	1
	NCI-H322m	0.8		T47 D	0.4
	NCI-H522	0.6			
	HOP-62	0.6			

Renal cancer	A498	79.4	CNS cacner	SF539	6.0
	TK10	6.8		SF295	3.2
	CAKI-1	3.4		U251	1.4
	786-0	1.8		SNB75	0.6
	RXF393	1.6		SNB19	0.4
	ACHN	0.8		SF268	0.4
	UO31	0.6			
	SN12C	0.2			
			Ovarian cancer	SK-OV3	15.8
				Ovcar5	1.8
				IGROV1	0.8
				OVCAR14	0.6
				OVCAR8	0.4
				OVCAR3	0.4

hPGFS expression in different cell lines was normalised from the expression of NCM425, a normal colon mucosa cell line.

).

### Expression of hPGFS was detected primarily in tumour cell lines from respiratory and digestive organs

**Figure 4:**
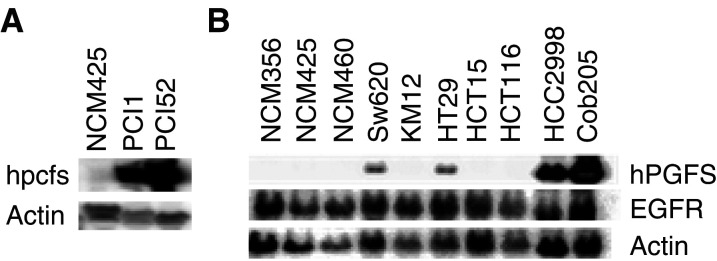
Northern blot analysis of gene expression in cell lines derived from normal mucosa and tumours of SCCHN and colon. NCM356, NCM425 and NCM460 are normal mucosal cell lines. COLO205, HCC2998, HT29, KM126, SW620, HCT15 and HCT116 are colon tumour cell lines. PCI52 and PCI1 are tumour cell lines of SCCHN. (**A**) Increased expression of hPGFS in SCCHN tumour cell lines. (**B**) Elevated expression of hPGFS in colon tumour cell lines. Actin was used as an internal control.

To verify that increased expression of hPGFS in primary SCCHN tumours was at least partially due to the increase in tumour-cell expression, we analysed hPGFS expression in five SCCHN cell lines, and 59 tumour cell lines derived from tumours from other organs, including breast, prostate, renal, colon, lung, blood, central nervous system, ovary, and skin. Three normal mucosal cell lines, NCM356, NCM425, and NCM460, were used to compare the relevant level of hPGFS expression. The expression of hPGFS in tumour cell lines was defined as more than a two-fold increase over the level of the control cell line NCM425. As demonstrated by Northern blot results, in Figure 4

and Table 3, the level of hPGFS was increased 8.6- to 74.5-fold in four out of five cell lines from SCCHN; 2.1- to 105-fold in five out of seven cell lines from colons. Thus, the increased expression of hPGFS detected in tumours (Figure 3, Table 2) was at least partially due to its upregulation in tumour cells (Figure 4, Table 3). Interestingly, hPGFS was not detected in melanoma (zero out of nine) cell lines, but was detected in a small proportion of tumour cell lines derived from breast, central nervous system, ovarian, leukaemia, and renal cancer (Table 3). The magnitude of increase in hPGFS expression in these tumour cell lines was also low. However, hPGFS was significantly increased in tumour cell lines derived from colon cancer, Non-small-cell (NSC) lung cancer, head and neck cancer. Up to a 500-fold increase of hPGFS expression was detected (Table 3), suggesting that the high level of hPGFS expression was associated with tumour types.

## DISCUSSION

Using a modified DD-PCR technique, in combination with analysis of DNA sequence and gene expression by bioinformatics and Northern blot respectively, we made two interesting observations. One revelation was the downregulation of an apoptosis candidate gene, *sFRP*, and a tumorigenesis suppressor gene, *DSG3*, in SCCHN that are also found by other investigators in other tumour tissues (Finch *et al*, 1997; Zhou *et al*, 1998), demonstrating that different tumours may share a common molecular mechanism in tumorigenesis. The other was discovering the linkage of increased expression of hPGFS to multiple tumours. The increased expression of hPGFS may link to tumorigenesis and maybe able to serve as a potential molecular marker and drug target as supported by three lines of evidence discussed below.

The first line of evidence came from the biochemical function of hPGFS. *hPGFS* was cloned from a human lung cDNA library with the use of bovine PGF in 1999. The sequence analysis indicated that this cDNA matched *KIAA0119*, cloned from the human immature myeloid cell line KG-1 in 1995 (Nagase *et al*, 1995). Although the biological function of KIAA0119 is not clear, its biochemical function might serve as a steroid dehydrogenase. The deduced amino-acid sequence was identical to that of AKR1C3, except for two amino acids (Khanna *et al*, 1995). The enzymatic study demonstrated that the natural substrates of hPGFS were PGD2 and PGF2, but not steroid hormones such as dihydrotestosterone, catalysed by AKR1C3 (Suzuki-Yamamoto *et al*, 1999). It has been known that cyclooxygenase 2 (Cox2) is overexpressed in head and neck tumour tissues, induces tumorigenesis, and modulates the production of PGD2 (Kawamori *et al*, 1998). Thus, these two enzymes are in the same biochemical pathway and they may coordinate with each other in promoting tumorigenesis by promoting production of different PGs. PGs have been known to favour tumorigenesis (Subbaramaiah *et al*, 1997) through immunosuppression and increasing neovascularisation (Nugent *et al*, 1996). However, the exact roles of PGFS and PGD2 in tumorigenesis have not been studied yet. The other PGD downstream molecule, PGE, is known to suppress the immune system, which may promote tumorigenesis. We speculate this may also be the case for PGF2.

The second line of evidence to support increased expression of hPGFS is a potential tumour marker and a drug target that came from the wide expression of this gene in tumour cell lines from many different tumour types. Interestingly, the expression of hPGFS was preferentially increased in tumour cell lines derived from tumours located in respiratory and digestive organs. Up to a 500-fold increase in hPGFS expression was found in the tumour cell lines derived from the colon, lung, head and neck (Table 3). A higher frequency of elevated hPGFS expression in SCCHN tumours, compared to the frequency of increased EGFR expression, was found in this study (Table 2). However, the expression of hPGFS in tumour cell lines of melanoma, central nervous system, leukaemia, breast, renal, and ovarian cancer was either not detected or only modestly increased in a small portion of cell lines examined. Thus, hPGFS may also be a potential drug target for some of the tumours because the huge differential expression between some types of the tumours, in which a high level of expression occurs.

The third line of evidence to support the notion that increased hPGFS expression can be used as a potential molecular marker and a drug target is that hPGFS increased in a large number of primary tumour tissues. As shown in Table 2, hPGFS was increased in 10 out of 17 (59% *P*<0.05) larynx and hypopharynx SCC compared with negative surgical margins tissues. The overall frequency of elevated hPGFS expression in SCCHN tumours (40.5%) exceeds the frequency of EGFR (32.4%), a well-known molecular marker and drug target for multiple types of tumours. Moreover, these two markers may complement each other to make a better diagnosis than use of a single marker because the elevated expression of these two genes does not occur in the same tumour sample (Table 2). Combining hPGFS with EGFR and other tumour markers will certainly increase the accuracy of molecular diagnosis for specific type of tumours.

The magnitude of increased expression of hPGFS varied among different tumour tissues, despite the use of a consistent sampling procedure. One possibility is the heterogeneity in the tumour tissues from different patients. Microscopic laser capture of tumour-specific cells could avoid this problem, but it was impossible to get enough RNA from each of the 37 tumour tissues for Northern blot analysis. Although the PCR-based quantitation requires a less amount of RNA, the high homology of hPGFS with several other genes such as AKR1C3 (two amino acids difference) hindered this approach. The tissue heterogeneity-caused variation in hPGFS expression can be better examined when an hPGFS-specific antibody becomes commercially available.

In conclusion, this is the first demonstration that hPGFS is differentially expressed mainly in SCC of larynx and hypopharynx, as well as tumour cell lines derived from lung and colon cancer. Therefore, it may be a potential therapeutic target for these cancers.
